# mTORC1 sustains vision in retinitis pigmentosa

**DOI:** 10.18632/oncotarget.4466

**Published:** 2015-06-14

**Authors:** Lolita Petit, Claudio Punzo

**Affiliations:** Department of Ophthalmology and Gene Therapy Center, University of Massachusetts Medical School, Worcester, MA, USA

Photoreceptors are highly specialized neurons that have evolved to optimally capture photons. Understanding their demise, in order to develop therapeutic strategies, has the potential to improve the quality of life of millions of patients.

Retinitis pigmentosa (RP) is the most frequent form of inherited photoreceptor degeneration. In many cases, the genetic defects are specific to the night active rod photoreceptors; however, cones, which mediate daytime and high acuity vision, die as well [1]. The dependence of cones on rods has puzzled the field of retinal biology for decades. Many hypotheses have been proposed that are supported by experimental evidence; however, the delay in cone death has been minimal and short-lived.

We recently proposed a new model where cone loss is caused by a nutrient shortage (particularly glucose) induced by the collapse of the retinal architecture as rods die [2]. In the retina, rods and cones are densely packed into the same layer. Because rods account for over 95% of photoreceptors, their loss profoundly affects the retinal structure making cones vulnerable. Our hypothesis for cone loss was based on the findings that cones increase the expression of metabolic genes at the onset of cone death, and display signs of prolonged starvation during degeneration. These changes were accompanied by changes in the insulin/mammalian target of rapamycin (mTOR) pathway, a pathway that regulates cell metabolism by balancing demand with supply. To test the role of this pathway during cone degeneration, we treated the retinal degeneration-1 mouse model of RP with daily systemic injections of insulin. Similar to the experimental evidence of previous hypotheses the effects of insulin were not long lasting, albeit substantial. This short-lived effect may have been caused by the feedback loop within the pathway, which causes cells to become insulin resistant over time [3].

To circumvent the feedback-loop, test if insulin acted directly on cones, and test the long-term therapeutic potential of the pathway on cone survival we took a genetic approach using various conditional alleles of genes downstream of the insulin receptor, that when deleted either increase or ablate mTOR activity. Deletion in cones of the two negative regulators of mTOR, the phosphatase and tensin homologue (*Pten*) and the tuberous sclerosis complex 1 (*Tsc1*), both significantly prolonged cone survival while deletion of *Raptor*, the accessory protein of the mTOR complex 1 (mTORC1), accelerated cone death during disease [3, 4]. Concomitant loss of *Pten* and *Raptor*, as well as *Tsc1* and *Raptor*, and *Tsc1* and *Rictor*, the mTORC2 accessory protein, confirmed that the insulin-mediated effect on cone survival was indeed driven by mTORC1. Because the effect on cone survival upon loss of *Tsc1* was the most robust and long lasting described thus far, the data supports the idea that nutrient shortage may be the overarching problem cones face as rods die. This can be explained by the high-energy demand of photoreceptors. In a healthy retina in addition to re-equilibrating membrane potentials photoreceptors need to synthesize membranes and proteins at a rate equivalent to one cell division per day, as these are lost in the daily shedding of their photosensitive structure [5]. Additionally, photoreceptors need to synthesize large quantities of NADPH to neutralize free radicals produced by incident light and the high metabolic rate, making them one of the highest energy consuming cells in the body [6].

To sustain their metabolic needs photoreceptors have a high glycolytic rate that is accompanied by secretion of lactate in the presence of ample oxygen, a phenomenon known as the Warburg effect and typically found in cancer cells. Like proliferating cells, photoreceptors thus express high levels of hexokinase II and pyruvate kinase-2. In dividing cells these genes are regulated by mTORC1, which controls a wide range of metabolic pathways including glycolysis, fatty acid synthesis and the pentose phosphate pathway. It is thus not surprising that loss of *Tsc1* increased the expression of these genes in cones in addition to other metabolic genes [4]. Therefore, loss of *Tsc1* may have promoted cone survival by improving glucose uptake, retention, and utilization. Interestingly, all of these genes appeared already upregulated in cones during disease, albeit to a lesser extend. Loss of *Tsc1* further increased their expression making cones more resistant to the nutrient shortage. Consistent with that, loss of mTORC1 activity during disease accelerated cone death, as cones fail to balance demand with supply, while combined loss of *Tsc1* and mTORC1 not only failed to promote cone survival but resulted in the same acceleration of cone death as seen upon loss of mTORC1 alone. In contrast, loss of mTORC1 in wild-type cones had no effect suggesting that mTORC1 is mainly required when the metabolic equilibrium is disturbed [7].

To test if loss of *Tsc1* can promote cone survival in other models of RP, we repeated the experiments in the slow progressing rhodopsin knockout mouse. Cone death was significantly delayed in this model too, indicating that increasing mTORC1 activity has broad therapeutic implications for RP [4]. While many mTORC1 inhibitors have been identified due to their ability to reduce proliferation in cancers our findings highlight the need to identify activators of mTORC1 that could be beneficial for other diseases.
